# Supplementary material for: Mapping specificity, cleavage entropy, allosteric changes and substrates of blood proteases in a high-throughput screen
Source: Nat Commun. 2021 Mar 16;12:1693. doi: 10.1038/s41467-021-21754-8 (PMC7966775; doi:10.1038/s41467-021-21754-8)
Supplement: Supplementary file 1 — Supplementary Information [file 41467_2021_21754_MOESM1_ESM.pdf]

Mapping specificity, cleavage entropy, allosteric changes and substrates of blood proteases in a high-throughput screen

Federico Uliana, Matej Vizovišek, et al.

Supplementary Information

Supplementary Figure 1

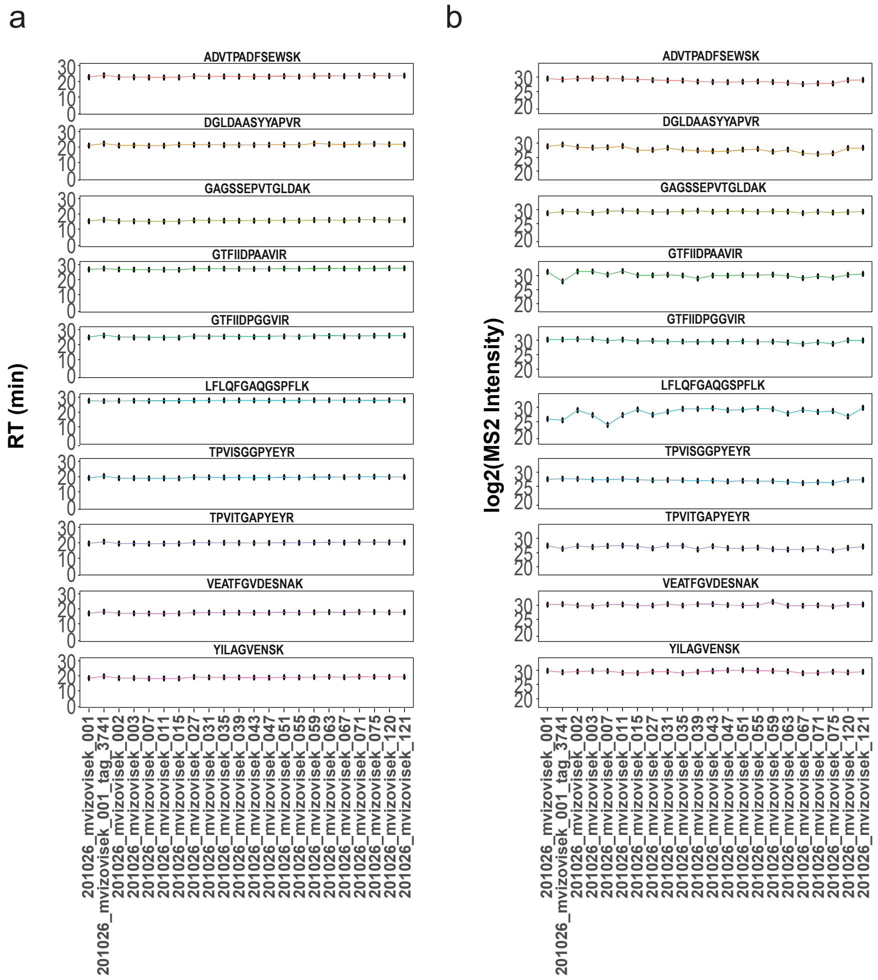

Supplementary Figure 1. Quality control of the LC-MS runs with iRT peptides.

The performance of the LC-MS was monitored with targeted analysis of iRT peptides used as an external standard after every triplicate measurement. The column performance and the sensitivity of the instrument were checked by monitoring the (a) retention time and (b) fragment MS2 intensities.

Supplementary Figure 2

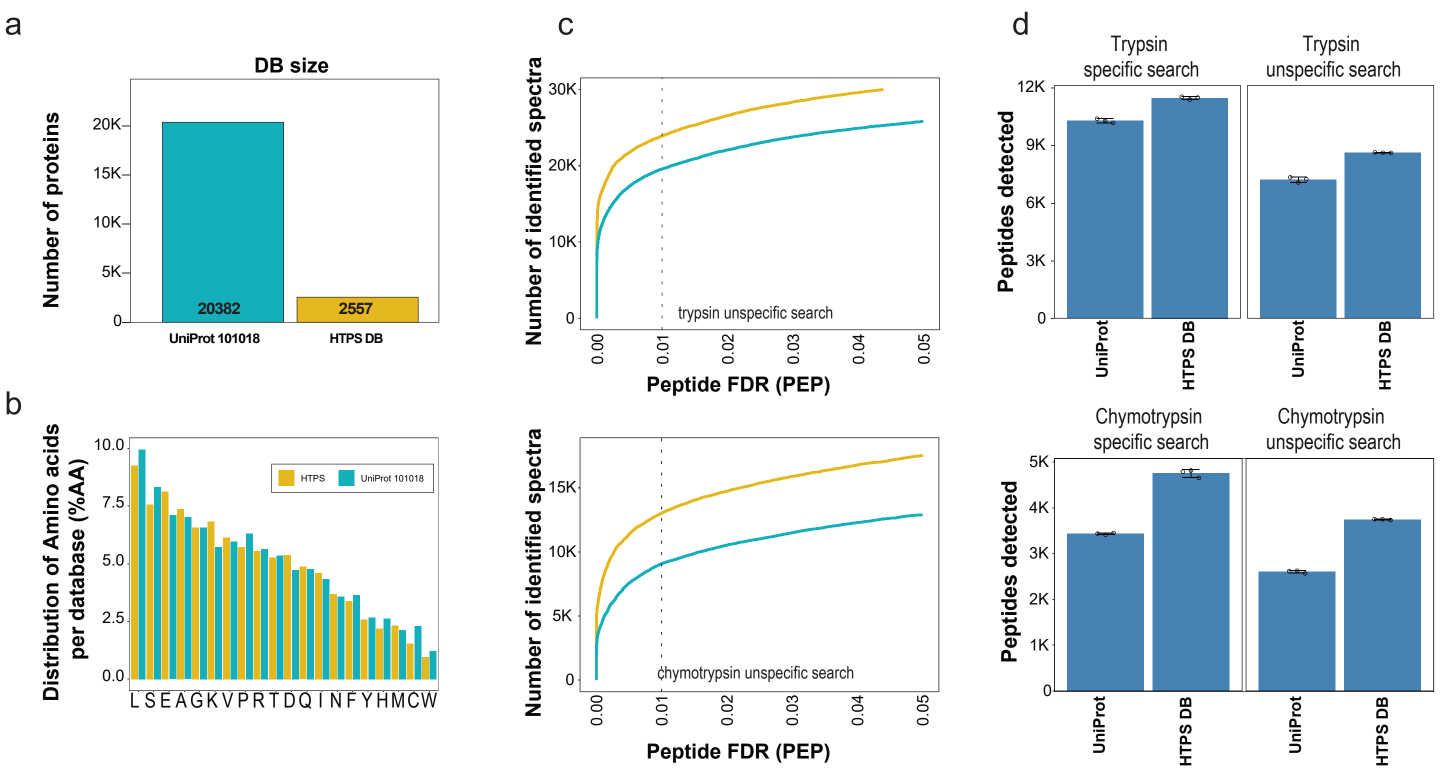

Supplementary Figure 2. Target database generation for HTPS and evaluation of performance for specific and unspecific database searches.

(a) The database size (HTPS_DB.fasta) focused on the abundant proteome covering around 12% of the human UniProt Homo sapiens database (2557 proteins). (b) Distribution of amino acid residues abundance in UniProt database and in HTPS_DB.fasta database. (c) Distribution of peptide FDR for identified peptides using specific and unspecific searching mode in MaxQuant for UniProt database (blue line) and HTPS_DB.fasta (yellow line). The analysis was performed for a sample generated by Trypsin (upper panel) and Chymotrypsin proteolysis (lower panel). (d) Numbers of identified peptides (FDR < 0.01) for unspecific and specific searches using UniProt database and HTPS_DB.fasta database. The analysis was performed for samples generated by Trypsin (upper panel) and Chymotrypsin (lower panel) proteolysis (n=3 independent replicates). Data are presented as mean values +/- SD.

Supplementary Figure 3

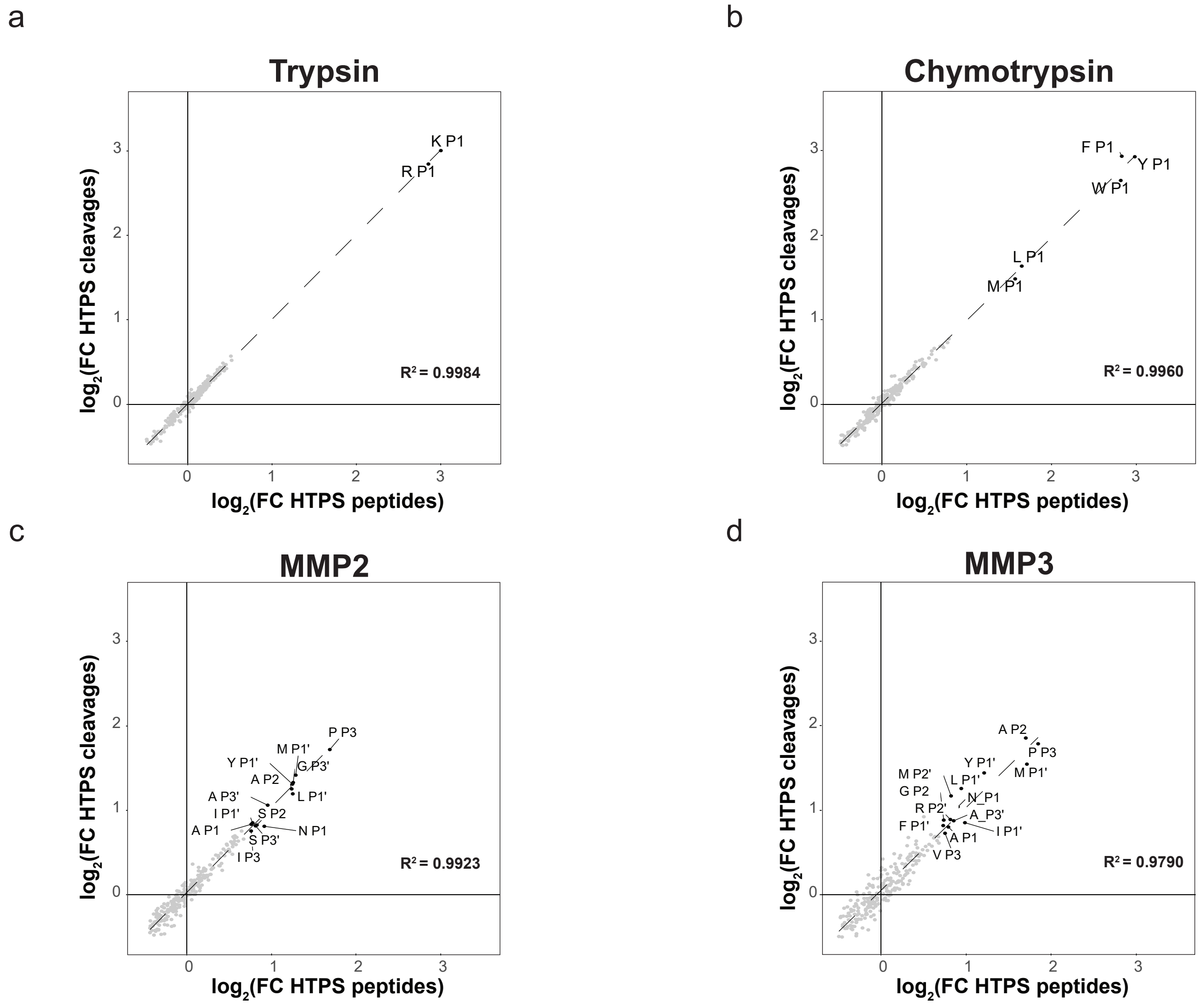

Supplementary Figure 3. Influence of original peptide termini on the determined specificity profiles.

Correlation of the log_2FC enrichment of detected substrate preference for (a) Trypsin, (b) Chymotrypsin, (c) MMP2 and (d) MMP3 generated from all detected peptide termini (FC HTPS peptides) and from cleavage sequences (FC HTPS cleavages).

Supplementary Figure 4

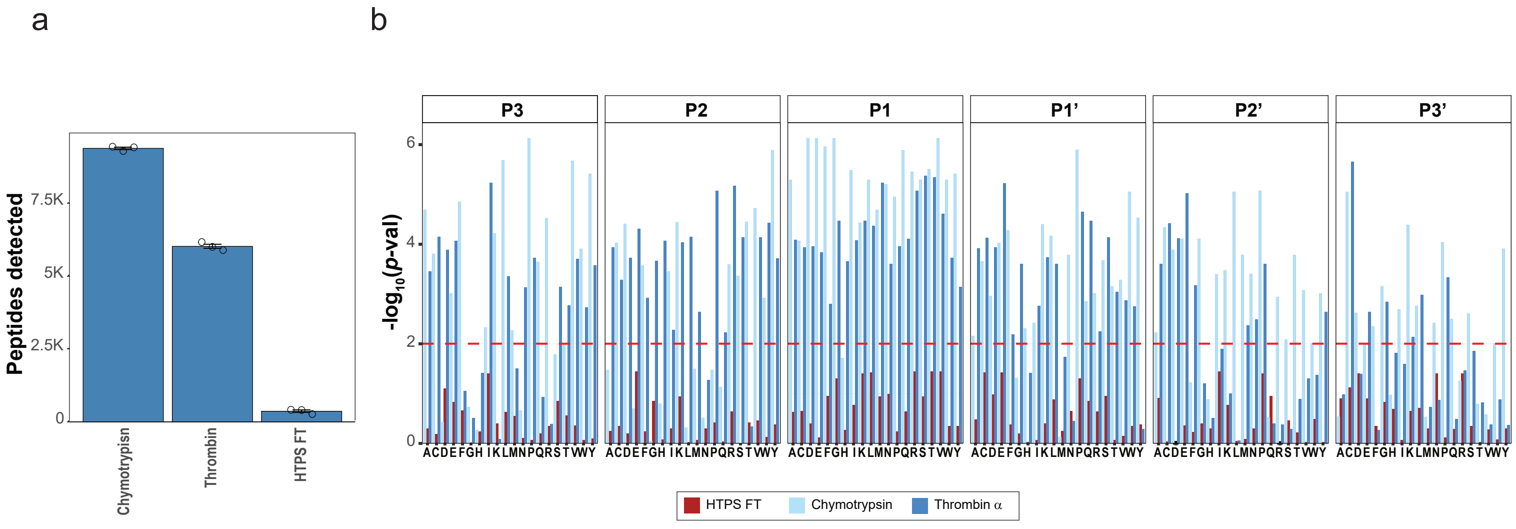

Supplementary Figure 4. Evaluation of the proteolytic background and its impact on specificity. (a) Number of peptides detected after proteolyzing the native cell lysate with Chymotrypsin, α-Thrombin or without proteolysis, i.e. HTPS flow through (HTPS FT) (n=3 independent replicates). Data are presented as mean values +/- SD. (b) Positional amino acid enrichments for Chymotrypsin, α-Thrombin or without proteolysis as determined from the respective positional *p*-values from paired two-sided t-test. Importantly, no significant enrichment was observed in the HTPS FT samples suggesting that proteolytic background does not have an impact on the determined specificity patterns.

Supplementary Figure 5

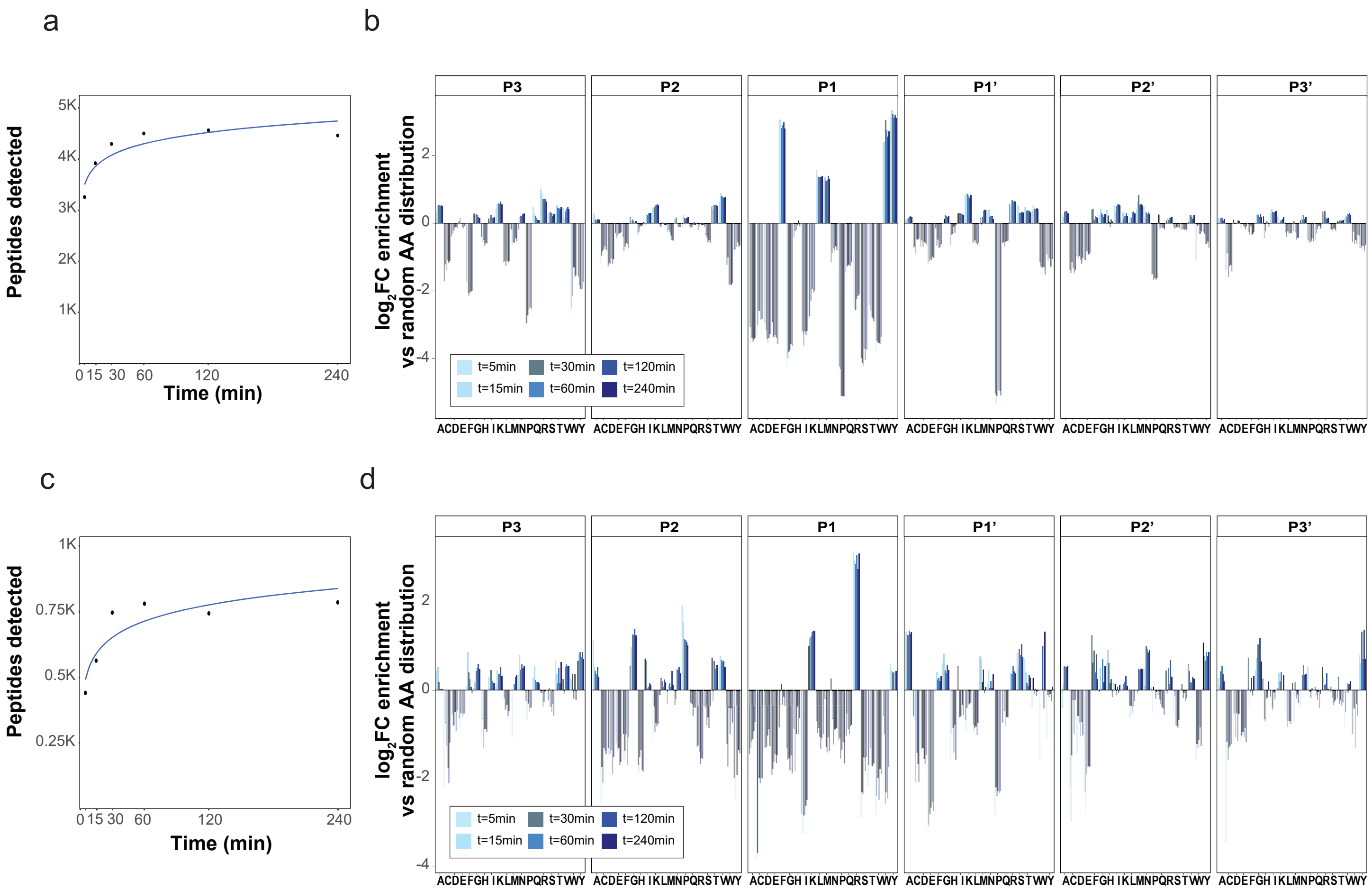

Supplementary Figure 5. The peptides identified with HTPS for 0 – 240 min proteolysis with Chymotrypsin or α-Thrombin and the impact of cleavage numbers on the protease specificity profile.

The number of peptides identified by HTPS for 0 – 240 min proteolysis with Chymotrypsin (a) and α-Thrombin (c). Chymotrypsin (b) and α-Thrombin (d) specificity profile is not influenced by the number of detected cleavage products over time.

Supplementary Figure 6

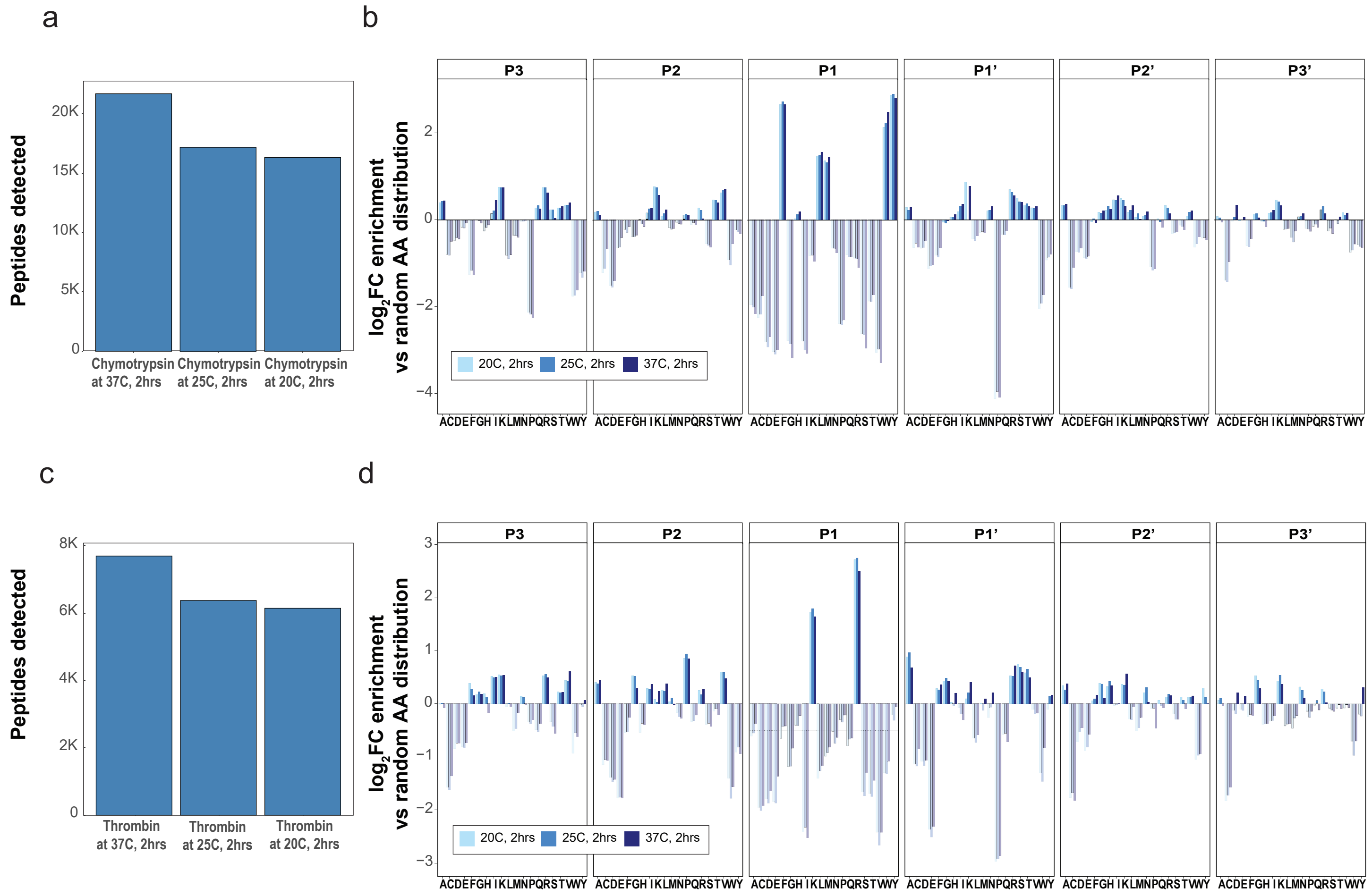

Supplementary Figure 6. Temperature-dependence of proteolysis with Chymotrypsin and α-Thrombin and the respective specificity profiles.

The number of peptides identified by HTPS provides at 37 °C, 25 °C and 20 °C after 2 hour proteolysis with Chymotrypsin (a) and α-Thrombin (c) (n=3 independent replicates). The specificity profiles of Chymotrypsin (b) and α-Thrombin (d) are not influenced by the number of detected cleavage products at the investigated temperatures (n=3 independent replicates).

Supplementary Figure 7

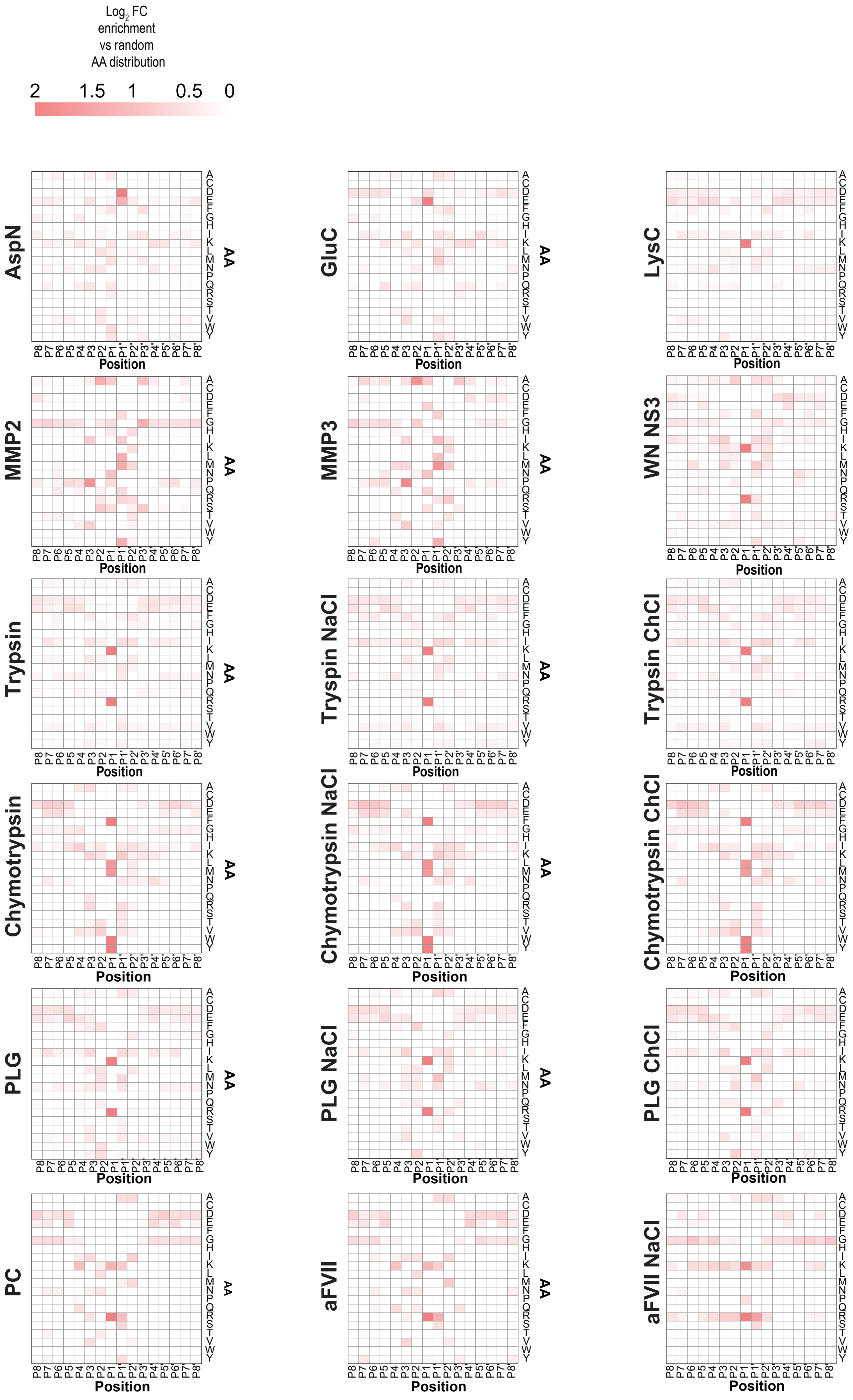

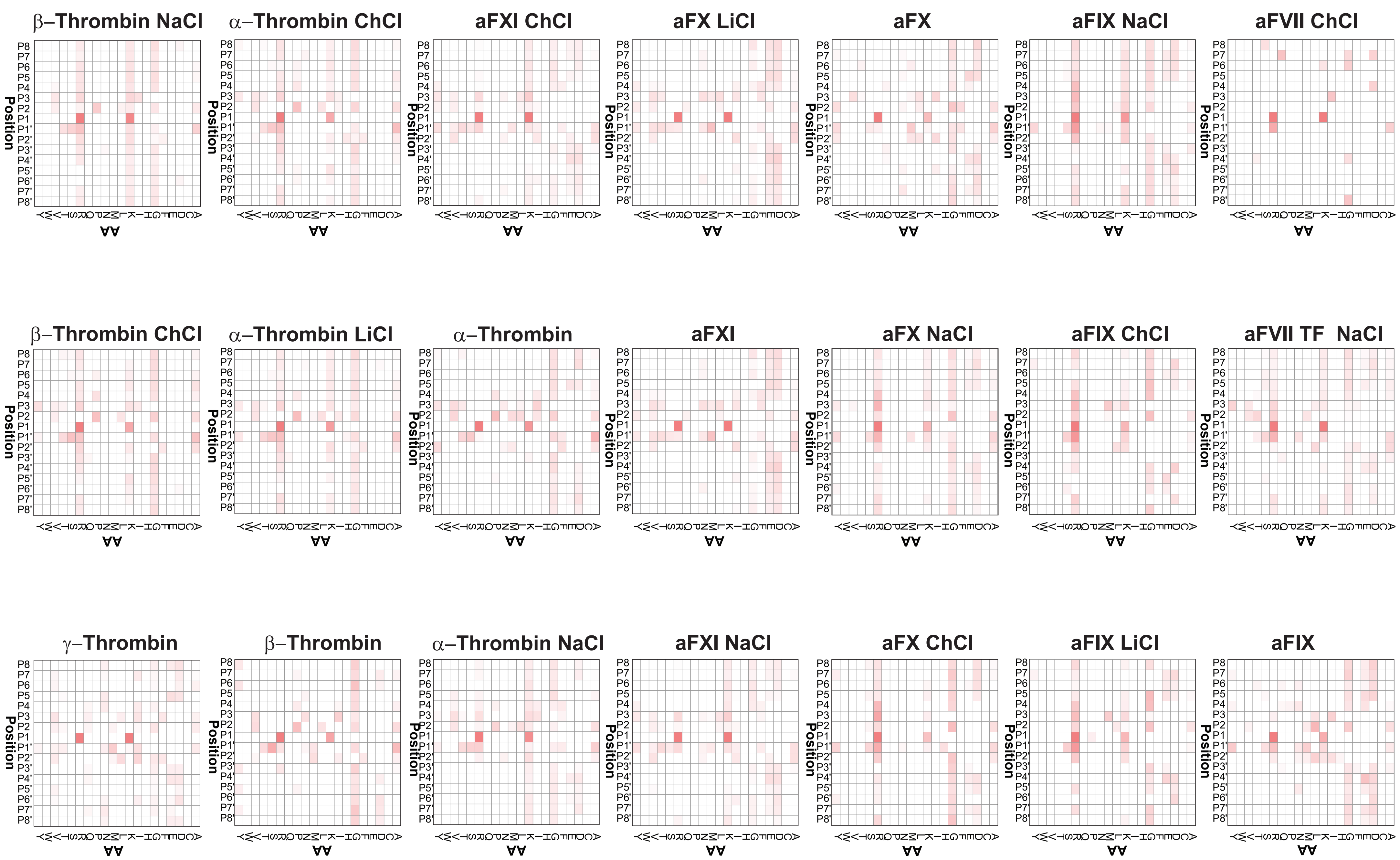

β−Thrombin NaCl
α−Thrombin ChCl
aFXI ChCl
aFX LiCl
aFX
aFIX NaCl
aFVII ChCl
β−Thrombin ChCl
α−Thrombin LiCl
α−Thrombin
aFXI
aFX NaCl
aFIX ChCl
aFVII TF NaCl
γ−Thrombin
β−Thrombin
α−Thrombin NaCl
aFXI NaCl
aFX ChCl
aFIX LiCl
aFIX
Position
P8 P7 P6 P5 P4 P3 P2 P1 P1' P2' P3' P4' P5' P6' P7' P8'
AA
A C D E F G H I K L M N P Q R S T V W Y

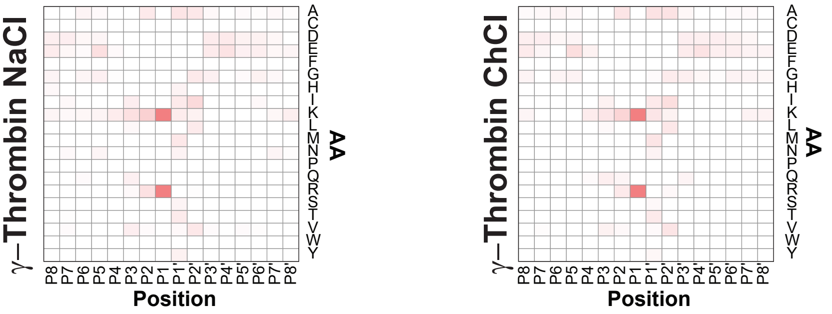

Supplementary Figure 7. Heat maps of positional substrate preferences of blood cascade proteases.

The heatmaps show the significant log_2FC enrichment for amino acids at investigated positions (P8-P8') in comparison to a random distribution. The respective natural amino acids are sorted alphabetically.

Supplementary Figure 8

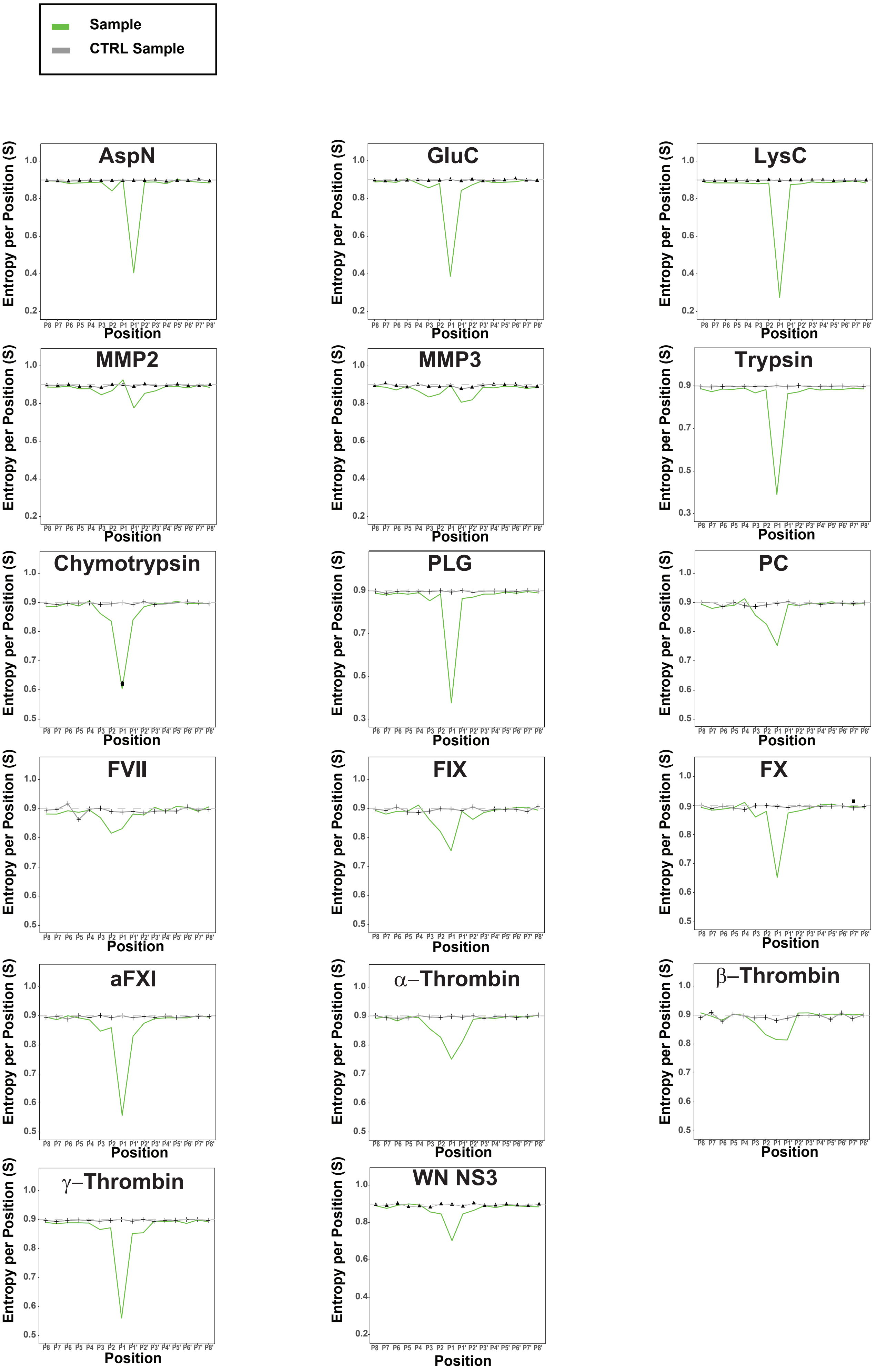

Supplementary Figure 8. Positional entropy profile plots of studied proteases.

The plots show positional entropy values (S) for each position (P8-P8') for all proteases included in the HTPS screen calculated according to Fuchs et al.[34]

Supplementary Figure 9

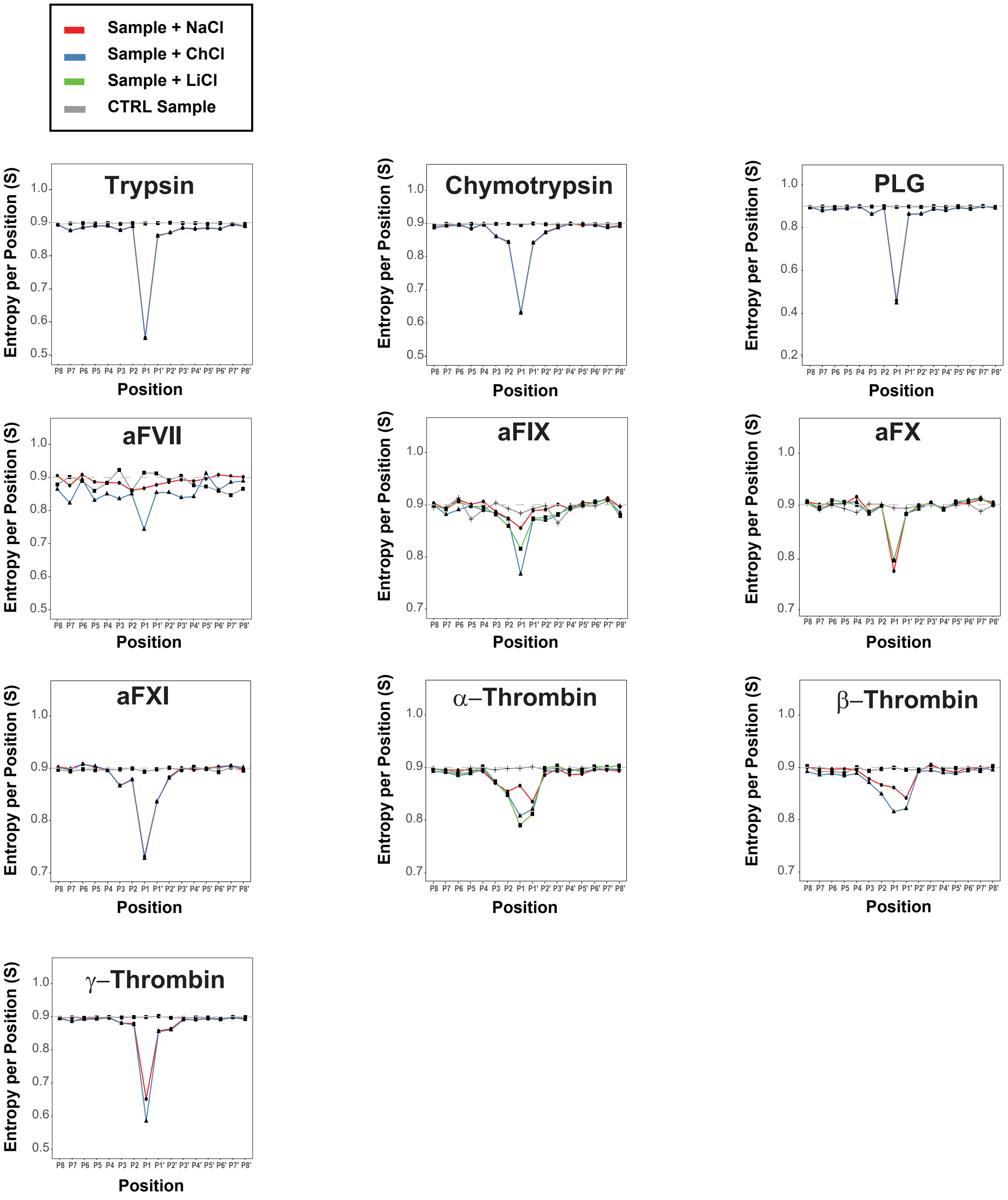

Supplementary Figure 9. Positional differential entropy profile plots of studied proteases.
The plots show positional entropy values (S) for each position (P8-P8') for condition with NaCl (red), with ChCl (blue), LiCl (green) or for a control sample (grey) for all proteases included in the HTPS screen calculated according to Fuchs et al.[34]

Supplementary Figure 10

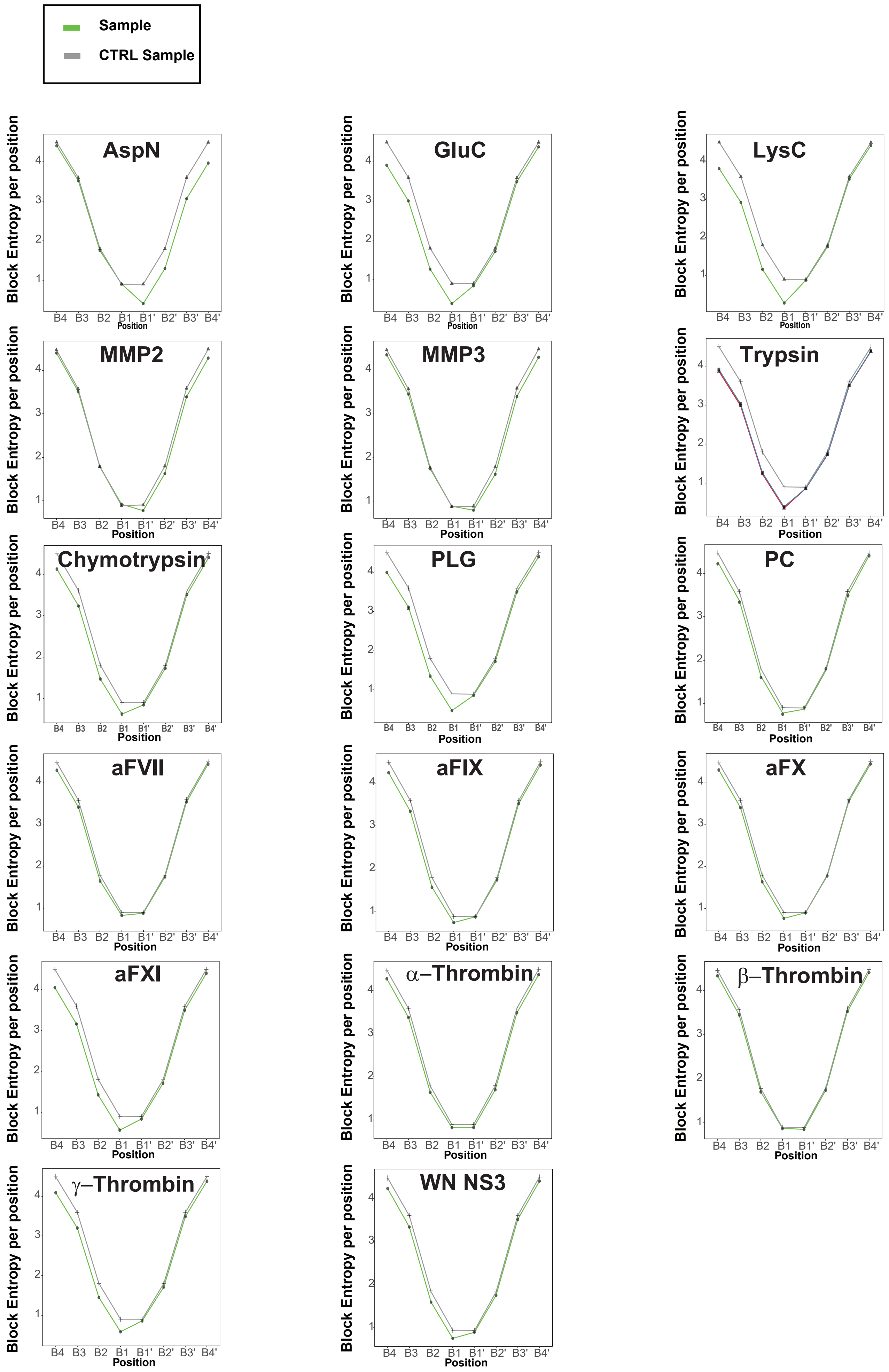

Supplementary Figure 10. Block entropy profile plots of studied proteases.

The plots show block entropy values for a block of amino acids where B1 includes position P1, B2 positions P2 and P1, B3 positions P3, P2 and P1 and complementary for the prime positions. The figures show (B4-B4') for samples (green) and controls (grey) as calculated according to Qi et al.[35]

Supplementary Figure 11

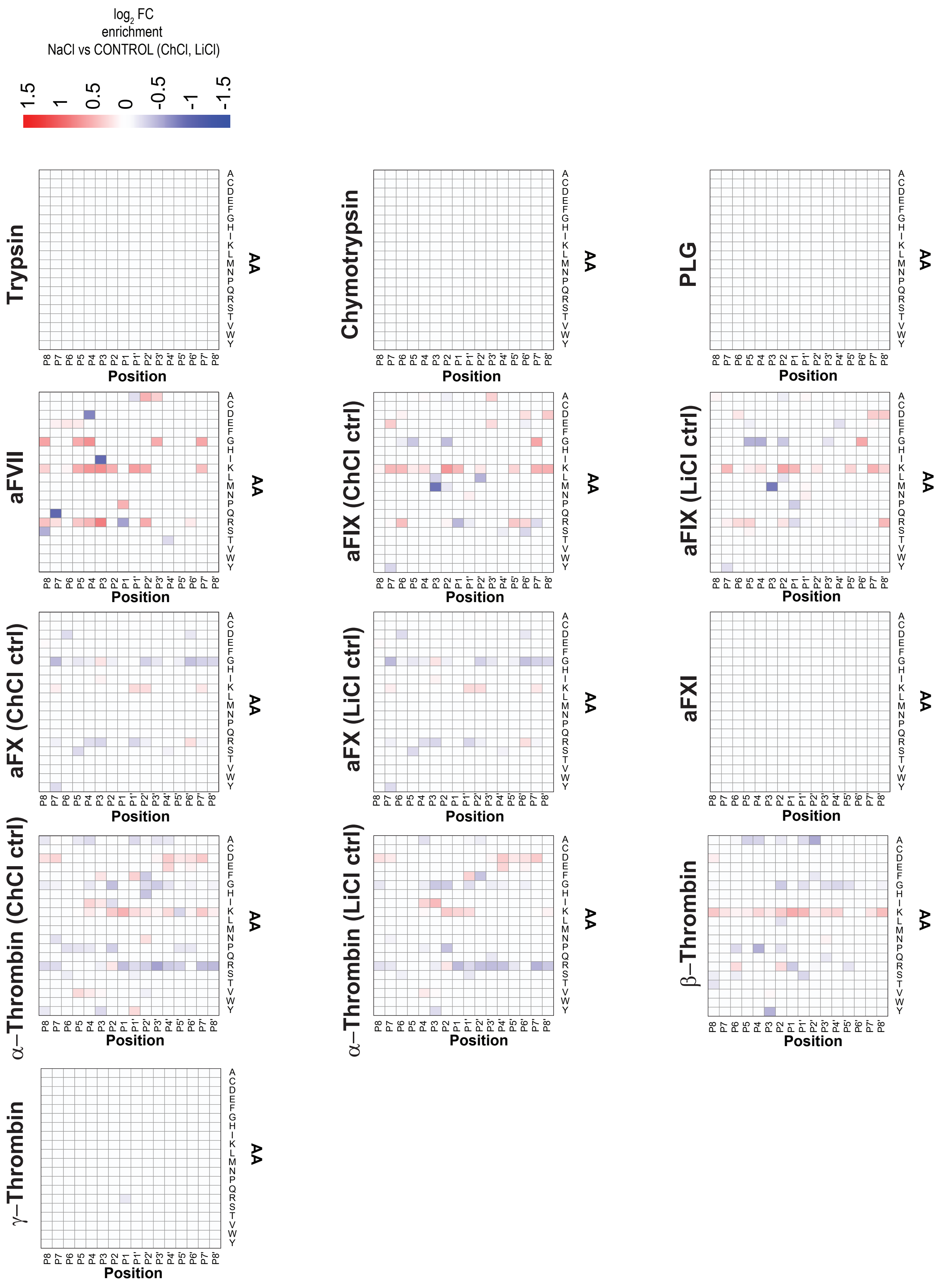

Supplementary Figure 11. Protease specificity changes as a result of Na^+ allosteric binding.

The positional specificity change of NaCl vs. ChCl/LiCl is shown for positions P8-P8' for all coagulation proteases included in the screen. The differences of significantly changing positional preferences are shown as the log_2FC for the tested conditions.

Supplementary Figure 12

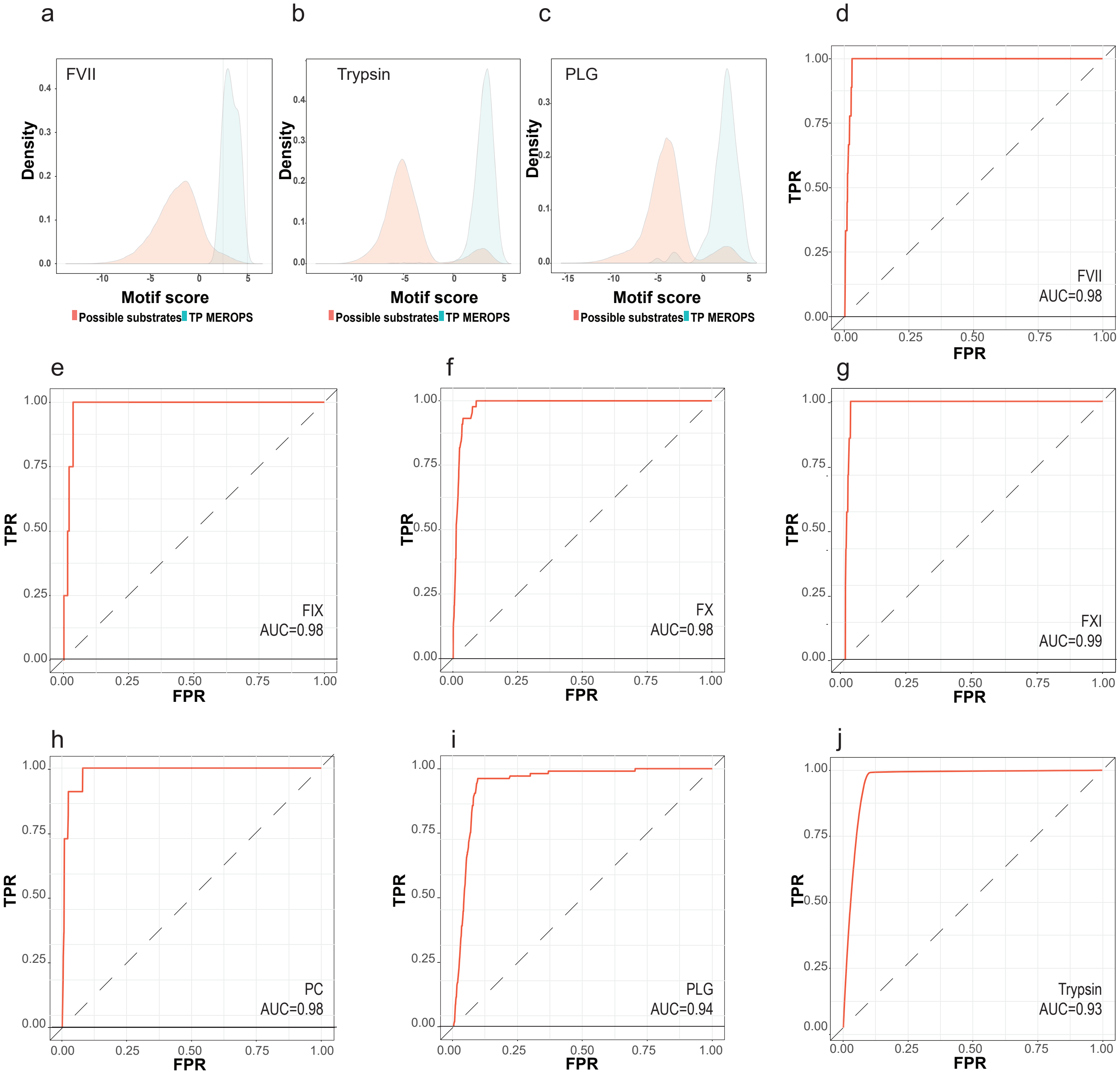

k

		After amino acid level filter					After protein level filter			
Protease	TP MEROPS	Threshold (FPR)	Motif score at 0.01 FPR	ID motifs	Precision (%)	Recall (%)	ID motifs	ID proteins	Precision (%)	Recall (%)
Thb	41	0.01	3.43	1385	1.88	63.41	878	289	2.90	56.10
FVII	8	0.01	3.43	1388	0.14	25.00	827	268	0.24	25.00
FIX	3	0.01	3.74	1387	0.07	33.33	840	277	0.12	33.33
FX	17	0.01	3.76	1478	0.20	17.65	871	270	0.23	11.76
FXI	6	0.01	3.70	1251	0.16	33.33	794	265	0.25	33.33
PC	8	0.01	3.47	1551	0.32	62.50	914	266	0.55	62.50
PLG	69	0.01	3.84	1213	0.82	14.49	818	259	1.22	14.49
**average**	**14**	**0.01**	**3.62**	**1379**	**0.51**	**35.67**	**848**	**270**	**0.78**	**33.78**

l

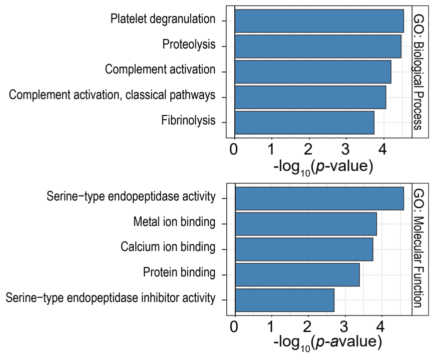

m

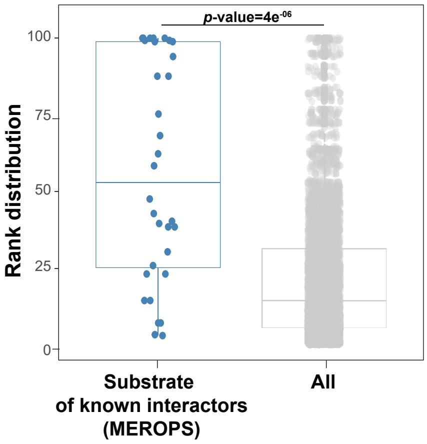

Supplementary Figure 12. Evaluation of the performance of three filtering steps for HTPS data to identify potential physiological substrates.

aFVII (a), Trypsin (b) and PLG (c) motif distribution (red) and true positive distribution (light blue) calculated from the positional enrichment of each amino acid of all secretome proteins against the HTPS Motif Score. (d-j) Receiver-operator curves (ROC) to evaluate the performance of the first filtering step using HTPS Motif Score and annotated substrates from MEROPS as the true positive set. (k) Summary table with statistical features (precision and recall) of different filtering steps applied at the level of amino acids or at the level of proteins. (l) GO analysis of biological processes and molecular functions of the proteins targeted by the blood cascade proteases calculated by DAVID[77] using the human secretome as background. (m) Distribution of protein rank for known (true positive) substrates annotated in MEROPS compared to all identified substrates. The score for MEROPS annotated substrates (light blue, median=53.02, n=32) and all substrates (light grey, median=14.59, n=4915). The boundaries of the box plot correspond to the quantiles Q1 (25%) and Q3 (75%). Lower and upper whiskers are defined by Q1 - 1.5IQR and Q3 + 1.5IQR. The *p*-value was calculated with a paired t-test.
