# Supplementary material for: Mapping specificity, cleavage entropy, allosteric changes and substrates of blood proteases in a high-throughput screen
Source: Nat Commun. 2021 Mar 16;12:1693. doi: 10.1038/s41467-021-21754-8 (PMC7966775; doi:10.1038/s41467-021-21754-8)
Supplement: Supplementary file 3 — Description of Additional Supplementary Files [file 41467_2021_21754_MOESM3_ESM.docx]

Description of Additional Supplementary Files

File Name: Supplementary Data 1

Description: Proteases included in the study with gene name, Uniprot accession and brief description are annotated. Results of the active site titration of blood coagulation proteases.

File Name: Supplementary Data 2

Description: Number of cleavages of proteases included in the study deposited in MEROPS. Comparison of cleavages detected by HTPS and the cleavages reported in MEROPS for proteases included in the study. The cleavages are reported either as numbers detected in a single condition or as numbers detected across multiple conditions (i.e. NaCl, ChCl, LiCl).

File Name: Supplementary Data 3

Description: Summary of the search settings used for specific and unspecific database searches, the corresponding numbers of identified MS and MS/MS spectra and the time needed to perform the searches.

File Name: Supplementary Data 4

Description: The table shows the detected cleavage sequences for the 3 investigated replicates covering positions P8-P8'.

File Name: Supplementary Data 5

Description: Positional amino acid enrichments for the proteases included in the study, positions P8-P8' covering 20 natural amino acids in alphabetical order (ID). All experiments were performed in triplicates, so for each protease there are 3 specificity columns (specificity_1, specificity_2, specificity_3) and 3 control (specificty_CTRL_1, specificity_CTRL_2, specificity_CTRL_3) columns with the calculated corresponding medians (median_specificity, median_CTRL_specificity). The median values were used to calculate the FC, p-value (pval) and the adjusted p-value (adj_p_val) with Benjamini-Hochberg correction. The FC significant shows a fold change positional enrichment with an adjusted p-value <0.01. The last column FC significant positive shows only positive fold change positional enrichment with an adjusted p-value <0.01.

File Name: Supplementary Data 6

Description: Positional cleavage entropies calculated for positions P8-P8' (ID). The entropy values were calculated for the investigated protease (entropy_1, entropy_2, entropy_3) and for the respective controls (entropy_CTRL_1, entropy_CTRL_2, entropy_CTRL_3) as a Shannon entropy calculation (Fuchs et al., 2013). The respective median entropies (median_entropy, median_CTRL_entropy) were used to calculate the positional fold of change (FC).

File Name: Supplementary Data 7

Description: Block entropy calculated for positions P4-P4' for the investigated proteases (protease) and the corresponding controls (ctrl). The differential block entropy per position (DELTA) comparing block entropies of proteases and the corresponding controls. The block entropy calculations were performed as described by Qi et al., 2017.

File Name: Supplementary Data 8

Description: Predicted substrates of investigated blood coagulation proteases (Gene name, Uniprot, Protein name) with the corresponding descriptions of Protein class, Biological process and Molecular function. Substrate peptide sequence covering P4-P4' positions (Peptide) with the corresponding start (start AA) and end (end AA) amino acids. Motif score of potential target sequences was generated from the detected significant fold changes for the target protease. Score Jpred predicts the solvent accessible regions that can be targeted by the investigated protease. Protein frequency of co-citation reports the number of instances where the respective proteins were cited together. Protein concentration in blood (pg/L) as reported in the Protein Atlas. Number of potential cleavage sites as predicted with the workflow. Ranking score calculated from co-citation frequency, number of identified protease substrates and concentration in the blood. The known substrates of coagulation cascade proteases are annotated in the True positive columns.

File Name: Supplementary Data 9

Description: Cleavages identified with reductive di-methylation strategy in C3 cleavage assay with Thrombin. High-confident cleavages (filter = yes) were defined by identification (PEP < 0.01) in all 3 treated samples but not in negative controls. Matching of high confident cleavages from in vitro cleavage assay with cleavages predicted with HTPS (Supplementary Data 8).
