# Supplementary material for: Mapping specificity, cleavage entropy, allosteric changes and substrates of blood proteases in a high-throughput screen
Source: Nat Commun. 2021 Mar 16;12:1693. doi: 10.1038/s41467-021-21754-8 (PMC7966775; doi:10.1038/s41467-021-21754-8)
Supplement: Supplementary file 13 — Reporting Summary [file 41467_2021_21754_MOESM13_ESM.pdf]

nature research

Corresponding author(s): Prof. Dr. Ruedi Aebersold

Last updated by author(s): Feb 4, 2021

Reporting Summary

Nature Research wishes to improve the reproducibility of the work that we publish. This form provides structure for consistency and transparency in reporting. For further information on Nature Research policies, see our Editorial Policies and the Editorial Policy Checklist.

Statistics

For all statistical analyses, confirm that the following items are present in the figure legend, table legend, main text, or Methods section.

n/a	Confirmed	
☐	☒	The exact sample size (n) for each experimental group/condition, given as a discrete number and unit of measurement
☐	☒	A statement on whether measurements were taken from distinct samples or whether the same sample was measured repeatedly
☐	☒	The statistical test(s) used AND whether they are one- or two-sided<br>*Only common tests should be described solely by name; describe more complex techniques in the Methods section.*
☒	☐	A description of all covariates tested
☐	☒	A description of any assumptions or corrections, such as tests of normality and adjustment for multiple comparisons
☒	☐	A full description of the statistical parameters including central tendency (e.g. means) or other basic estimates (e.g. regression coefficient) AND variation (e.g. standard deviation) or associated estimates of uncertainty (e.g. confidence intervals)
☐	☒	For null hypothesis testing, the test statistic (e.g. F, t, r) with confidence intervals, effect sizes, degrees of freedom and P value noted<br>*Give P values as exact values whenever suitable.*
☒	☐	For Bayesian analysis, information on the choice of priors and Markov chain Monte Carlo settings
☐	☒	For hierarchical and complex designs, identification of the appropriate level for tests and full reporting of outcomes
☐	☒	Estimates of effect sizes (e.g. Cohen's d, Pearson's r), indicating how they were calculated

Our web collection on statistics for biologists contains articles on many of the points above.

Software and code

Policy information about availability of computer code

Data collection	Thermo Fischer Xcalibur was used to record the data on Orbitrap Elite (version 3.0.63, Thermo Fischer Scientific) and Orbiprap Fusion (4.3.73.11, Thermo Fischer Scientific) mass spectrometers.
Data analysis	All database searches were performed with MaxQuant version 1.5.2.8. Analysis of MaxQuant results was performed with a series of in-house scripts deposited on Github (https://github.com/anfoss/HTPS_workflow) under MIT license. Analysis of PRM measurements was performed with Skyline-daily (version 20.1). For data visualization, R (version 3.4.3) was used. IceLogo (version -1.2) was used for visualization of protease specificity. Analysis of cleavage kinetics data was performed with GraphPad Prism 8.<br>Molecular docking was performed with HPEPDOCK server (http://huanglab.phys.hust.edu.cn/hpepdock/). For visualization of docking solutions PyMOL (version 0.99rc6) was used. Amino acid exposition was calculated with JPred4 tool (http://www.compbio.dundee.ac.uk/jpred/). GO analysis was performed with David (version 6.8). Network visualization was done with Cytoscape (version 3.8.0).

For manuscripts utilizing custom algorithms or software that are central to the research but not yet described in published literature, software must be made available to editors and reviewers. We strongly encourage code deposition in a community repository (e.g. GitHub). See the Nature Research guidelines for submitting code & software for further information.

Data

Policy information about availability of data

All manuscripts must include a data availability statement. This statement should provide the following information, where applicable:

- Accession codes, unique identifiers, or web links for publicly available datasets
- A list of figures that have associated raw data
- A description of any restrictions on data availability

The data is deposited to ProteomeXchange with the identifiers:

PXD018976 (protease profiling dataset and HTPS.fasta database), PXD020320 (PRM measurements of substrate peptides), PXD022959 (Allostery dataset for coagulation proteases), PXD022971 (Identification of Thrombin cleavage sites on C3), PXD022972 (Analysis of the HTPS FT; Kinetics of Thrombin and Chymotrypsin; Effect of temperature on Thrombin and Chymotrypsin) and PXD022973 (Characterization of WN NS3 and AspN). Human Uniprot database was downloaded from Uniprot (https://www.uniprot.org/). MEROPS protease substrate database was downloaded from the MEROPS web page (https://www.ebi.ac.uk/merops/). Secretome data was obtained from Protein Atlas (https://www.proteinatlas.org/). The code and the example dataset to run the data analysis functions is available from GitHub (https://github.com/anfoss/HTPS_workflow).

Field-specific reporting

Please select the one below that is the best fit for your research. If you are not sure, read the appropriate sections before making your selection.

- [x] Life sciences
- [ ] Behavioural & social sciences
- [ ] Ecological, evolutionary & environmental sciences

For a reference copy of the document with all sections, see nature.com/documents/nr-reporting-summary-flat.pdf

Life sciences study design

All studies must disclose on these points even when the disclosure is negative.

Sample size	Sample size calculation was not performed. For the sample size we consider 3 independent replicates to perform statistical test.
Data exclusions	No data was excluded from the main analysis. For the revision experiments performed at 20°C we excluded Protein C from the data analysis because of insufficient activity.
Replication	All protease characterizations except for WN NS3 were performed in three independent replicates (n=3). All attempts of replication were successful. Due to the lower amount of protease available the experiment with the WN NS3 protease was performed once (n=1).
Randomization	Samples were randomized for sample preparation in each experiment. For the MS acquisition no randomization was performed, replicates were analyzed on the LC-MS system in blocks of 3 for each tested protease/condition. The performance of the instrument was closely monitored with iRT peptides to ensure consistent performance throughout the analysis (retention time and sensitivity).
Blinding	Data collection and analysis were not performed blinded. Samples were allocated to precisely-defined groups for the mesurements.

Reporting for specific materials, systems and methods

We require information from authors about some types of materials, experimental systems and methods used in many studies. Here, indicate whether each material, system or method listed is relevant to your study. If you are not sure if a list item applies to your research, read the appropriate section before selecting a response.

Materials & experimental systems

n/a	Involved in the study	
x		Antibodies
	x	Eukaryotic cell lines
x		Palaeontology and archaeology
x		Animals and other organisms
x		Human research participants
x		Clinical data
x		Dual use research of concern

Methods

n/a	Involved in the study	
x		ChIP-seq
x		Flow cytometry
x		MRI-based neuroimaging

Eukaryotic cell lines

Policy information about cell lines

Cell line source(s)	HEK293, ATCC CRL-1573. The cell line was obtained directly from ATCC.
Authentication	No additional authentication was performed.
Mycoplasma contamination	Cell lines were regularly tested for mycoplasma contamination. No contamination was detected.
Commonly misidentified lines (See ICLAC register)	None such cell lines were used in the study.

April 2020
